# The power of theory in the life sciences

**DOI:** 10.7554/eLife.112987

**Published:** 2026-09-18

**Authors:** Jacob L Fine, Finn Creeggan, Joshua Gertsvolf, Purav Gupta, Adam MR Groh

**Affiliations:** 1 Donnelly Centre, University of Toronto Toronto Canada; 2 Molecular Genetics, University of Toronto Toronto Canada; 3 Genome Biology and Bioinformatics, University of Toronto Toronto Canada; 4 Vector Institute Toronto Canada; 5 Arc Institute Palo Alto United States; 6 Montreal Neurological Institute-Hospital, McGill University Montréal Canada; 7 Integrated Program in Neuroscience, McGill University Montréal Canada

**Keywords:** theory, explanatory unification, philosophy of science, history of biology, computational biology, single-cell genomics, machine learning, scientific practice, None

## Abstract

The rapid growth of high-throughput biology and genomics over the past two decades has helped catalogue many different aspects of gene function in diverse cell types and conditions. More recently, advances in artificial intelligence and deep learning have shown tremendous promise in making accurate predictions of functional genomics measurements. These advances make it tempting to equate experimental cataloguing and accurate prediction with the growth of our theoretical understanding of biological processes – an equivalence we believe is ultimately misleading.

*“Theories are nets cast to catch what we call ‘the world’: to rationalize, to explain, and to master it. We endeavour to make the mesh ever finer and finer.”* Karl Popper (Popper, 1992).

Biology is often characterized as lacking a strong emphasis on theory, especially relative to physics. While this trope may apply to present-day experimental biology, we argue that a careful consideration of the power of theory in shaping past breakthroughs in biology offers a different perspective. Building on this historical foundation, we explore how theory can help address cell and molecular biology’s major challenges, particularly those that arise from the analysis of massive omics datasets and discuss how the practice of science itself may need to adapt for theory to assume this role.

## What is a theory?

The definition of theory has been a topic of considerable debate in the philosophy of science. Analytic philosophers of the mid-20^th^ century, including Karl Popper, Carl Hempel, and Thomas Nagel, largely agreed that theories are logical and structured statements, which formulate natural laws that enable explanation and prediction. Their main disagreement was about how theories should be evaluated. Popper, for instance, emphasized falsifiability, asserting that while theories can never be verified because an infinite number of experiments cannot prove a universal law, one refutation is sufficient to disprove a law (Popper, 1992). Conversely, Hempel and Nagel accepted the inductive argument that experimental evidence can be used to confirm a theory, rather than only falsifying it (Hempel, 1966; Nagel, 1961; Popper, 1992). Thomas Kuhn, on the other hand, was critical of the entire analytic concept of a theory, believing that scientists primarily work within accepted paradigms rather than testing current theories. Kuhnian paradigms are theoretical commitments that define legitimate problems and solutions, and importantly, are inextricably linked to the social influences of a particular period in scientific history (Kuhn, 1962). According to Kuhn, major scientific change is not purely logical but rather requires “paradigm shifts” during which scientific revolutions usher in new theoretical commitments.

In the 1980s, Philip Kitcher similarly softened previous analytic frameworks by proposing that good theories achieve explanatory unification – a process by which a small set of principles explain an increasingly large number of events (Kitcher, 1989). Soon after, Peter Godfrey-Smith conceptualized theories as models and heuristic frameworks that can be used to investigate the world (Godfrey-Smith, 2021). Both Kitcher and Godfrey-Smith saw theories as systems that organized explanations of many phenomena, rather than rigid, logical statements.

We do not attempt to resolve the longstanding philosophical debate about the nature of theory by proposing a universal definition. Instead, drawing primarily on Kuhn and Kitcher’s definitions of theory, we discuss how theory can function in biology to unify seemingly disparate observations under single interpretable frameworks that can help delimit empirical possibility to guide experimental and methodological design. Ultimately, this unification inherent to theory can harmonize observations into powerful explanatory frameworks and is essential to usher in novel scientific paradigms.

## Darwin, the theoretical biologist

Few examples illustrate the importance of theory in biology better than the work of Charles Darwin. While his meticulous fieldwork may be regarded as experimental in that he gathered extensive data to evaluate his hypotheses, such as performing crosses on plants and pigeons (Darwin, 1859), Darwin’s main insights into the underlying mechanisms of selection and adaptation were not primarily the result of any specific experiment or direct perturbation. As Ernst Mayr later argued, Darwin’s theory of descent with modification driven by natural selection did not represent an individual discovery, but rather a “novel integration of previously established facts” (Mayr, 1982). Thus, while lauded by experimental biologists, Darwin’s achievement in *On the Origin of Species* is perhaps best understood as a monumental contribution to theoretical biology.

Indeed, Darwin’s theory completely redefined how biological phenomena were explained. Before him, appeals to creation, ideal forms or a divine purpose was commonplace (Kitcher, 2003). Kitcher outlines how Darwin supplanted these appeals to religion with causal histories that traced the successive modifications of specific organismal groups – what he called “Darwinian histories” (Kitcher, 2003). Natural selection also epitomizes Kitcher’s explanatory unification principle, in that decades of ensuing observations and experiments in biogeography, embryology, and paleontology could be unified under the same set of evolutionary principles Darwin originally articulated (Kitcher, 1989). While the science of genetics would later explain the precise cellular mechanisms governing heredity, the theoretical scaffold guiding the construction of that great molecular edifice had already been built.

## Schrödinger and the information age of biology

A similar dynamic unfolded in the mid-twentieth century when biology again confronted fundamental questions about how information was transmitted from one generation to the next. To address this, physicist Erwin Schrödinger published *What Is Life?*, a concise yet highly influential book, which proposed that genetic information must be stored in a stable, “aperiodic crystal” capable of carrying a “code-script” (Schrödinger, 1944). This proposition, in tandem with the “Three-Man Paper” concerning gene structure published by Max Delbrück and colleagues in 1935 (Judson, 2011), was highly influential in biology because it represented a novel theoretical constraint and took influence from the physics-native concepts of energy and equilibrium. Importantly, Schrödinger offered no new biological data yet had a profound impact on the experiments that were subsequently conducted.

Crucially, Schrödinger’s work helped bring quantitative thinking into mainstream biology. Historians of molecular biology have documented the influence of Delbrück and Schrödinger’s ideas on a generation of physicists, including Francis Crick, who entered biology convinced that heredity must obey discernible physical principles (Judson, 2011; Kay, 2000). Horace Judson believed this event was “...the intellectual immigration that has mattered most to biology”, given its influence on the establishment of an era of biological thought concerned with finding fundamental mechanisms (Judson, 2011). Lily Kay was more critical of the direct correspondence between Schrödinger’s “code-script” and the later-established concepts of genetic inheritance but nevertheless believed that Schrödinger’s work brought a certain “cognitive legitimacy” and focus on information-based modes of explanation to biology that appealed to physical scientists (Kay, 2000). Therefore, although X-ray diffraction experiments were certainly critical to the discovery of the double helix in 1953, the model would not have been possible without a preceding paradigm shift in molecular biology that necessitated the importance of information-bearing macromolecules.

Schrödinger’s framing of inheritance in informational terms also helped establish a conceptual tradition in biology with parallels to Claude Shannon’s mathematical theory of communication (Shannon, 1948). This framework has since been applied to molecular biology via the quantification of information encoded within DNA-binding motifs (Schneider, 2006; Schneider et al., 1986) and the measurement of information flow through cellular signalling networks (Cheong et al., 2011; Tang and Hoffmann, 2022; Uda, 2020). Whether Shannon’s conceptualization of information transmission ultimately proves to be a fundamental organizing principle of living systems remains uncertain, but the history of molecular biology nevertheless illustrates how imported theoretical concepts can generate productive new ways of thinking.

## Theory in the age of cell-resolved computational biology

*“We still don’t understand how (genes and proteins) function together to create the smallest individual unit of life: the cell.”* Stephen Quake (Quake, 2024).

Quake’s observation captures a peculiar feature of contemporary biology. Cell-resolved genomics, spatial profiling, perturbational screens, and multimodal assays provide molecular descriptions of cells at a scale unimaginable even a decade ago. At the same time, machine learning models can predict gene expression from DNA sequence (Avsec et al., 2021; Linder et al., 2025), infer cellular responses to perturbation (Adduri et al., 2025), design proteins and genomes with desired properties (Brixi et al., 2026; Nguyen et al., 2024; Watson et al., 2023), and integrate diverse molecular measurements into unified representations of cellular state (Theodoris et al., 2023). And yet, despite such a capability to describe cells, we lack a fundamental account of how molecular interactions generate cellular identity and function.

This reality reflects a shift in the fundamental challenges facing biology. For much of biological history, progress was limited by access to observations. In today’s technology-driven landscape, the difficulty lies in determining whether common principles underlie the enormous diversity of measured cellular states. Returning to Kitcher once again, progress in biology may require a mass unification wherein scientists prioritize reducing the number of independent assumptions needed to explain a rapidly increasing set of observations (Kitcher, 1989).

While modern computational approaches are widely described as “data-driven”, theoretical commitments are applied at multiple levels throughout large-scale modelling and data processing efforts. Consequently, they operate as inductive biases that constrain model architecture, training procedures, optimization strategies, data selection and ultimately the functions that models learn. For example, one key inductive bias of convolutional neural networks (CNNs) is locality, namely, that physically close regions in the input space are sufficient to explain the output rather than long-range interactions (Liang et al., 2026). Attention-based architectures do not harbour this same inductive bias and are therefore better suited to evaluate long-range interactions (Vaswani et al., 2023). Understanding whether a biological phenomenon is influenced primarily by local or long-range interactions is one example of theory informing the inductive biases introduced into a modelling task. More generally, statistical models encode prior beliefs about the variables under study and their interactions (Pearl, 1986). Theory can therefore guide the selection of meaningful biological priors, constraining both the class of models considered and the space of solutions they are likely to learn.

By reducing the space of what is a priori possible, theory can also serve to constrain the space of meaningful biological hypotheses and therefore delimit the number of experiments for wet-lab biology. This is directly relevant to a well-known challenge in experimental biology: the complete number of possible cell states and molecular interactions defining biological systems vastly exceeds what can be reasonably assessed through experiment. Thus, even the most impressive cellular atlases capture only a small fraction of the empirical space occupied by living systems, and therefore, cannot survey the space of possible biological variation from which a model can meaningfully learn biological principles. ‘Learning’ in these conditions therefore depends critically on prior assumptions. While increasing scale may help large-scale models improve predictive accuracy, it does not eliminate the need to identify the most relevant molecular organizational principles of the system under study (Souza and Mehta, 2026).

This point becomes especially important in ongoing efforts to build virtual cells (Adduri et al., 2025; Chuai et al., 2026; Svensson et al., 2026). Such models aspire to represent cellular behaviour across a panoply of conditions and to predict the consequences of genetic or environmental perturbation. While their success will undoubtedly accelerate biological discovery, an accurate simulator does not reveal why particular cellular states emerge or which interventions are biologically meaningful (Dibaeinia et al., 2026). Put another way, a model may correctly predict a response while relying on statistical regularities that do not fully reflect the true biological mechanisms underlying that response (Breiman, 2001; Shmueli, 2010; Woodward, 2003). In this context, unexpected model failures may prove especially informative, revealing where current assumptions about cellular behaviour fall short, consistent with falsifiability being a key part of theory under Popper. Lastly, and perhaps most fundamentally, the utility of any virtual cell will always depend on the theoretical questions posed to it.

Thus, the challenge articulated by Quake is not solely a problem of computation but rather a problem of theory. We do not yet know which principles govern the emergence of cellular identity from molecular interactions, nor what form a quantitative theory of cellular organization will ultimately take. The extent to which such a framework will incorporate information theory, or an entirely new paradigm, if at all, remains unclear. What is apparent is the importance of theory-driven biology in shaping both the biological priors of present models and the questions posed to them in pursuit of this goal.

## Changes to the practice of biology

*“Seeking to be led by theory and knowledge will probably require shifts in research culture. Theorizing should be encouraged, and theories should be included in experimental papers to put data in context.”* Paul Nurse (Nurse, 2021).

If the goal of science is to understand the natural world, and the role of theory is to facilitate such understanding, then theory ought to play a central role in biology no less than in other sciences. However, contemporary biological research culture often privileges tightly scoped, hypothesis-driven projects or methodological scaling over more theoretical contributions. While these iterative approaches have yielded enormous advances, the historical cases of Darwin and Schrödinger suggest that transformative progress sometimes requires a different posture defined by sustained abstraction, cross-disciplinary fluency and a willingness to articulate principles before mechanisms are fully specified. We argue that this theoretical approach has traditionally helped reduce the space of possibility and unify knowledge to guide subsequent experimentation, while exposing anomalies within existing paradigms.

If we are to flood molecular biology with theory, it is important to consider how to embed the process of theory within the day-to-day reality of conducting biological science. François Jacob famously distinguished between “day science” and “night science” – the former referring to orderly testing of articulated hypotheses, and the latter, a more improvisational process through which new conceptual frameworks are born (Jacob, 1988). Taking influence from Jacob, Yanai and Lercher recently argued that rigid adherence to narrow hypotheses can obscure the discovery of unexpected structure in data (Yanai and Lercher, 2019; Yanai and Lercher, 2020). Formal modeling of how scientists choose experiments supports this notion; Dubova and colleagues suggest that theory-motivated science fixates too narrowly on confirming or disconfirming current hypotheses at the expense of exploratory approaches that are more likely to uncover unexpected patterns and thus generate improved theories (Dubova et al., 2022). Thus, in fields such as genomics and single-cell biology, where the structure of large datasets is only partially understood, premature theoretical commitment may channel attention toward what is easiest to explain rather than what is most informative. In this sense, although theory can function well to delimit biological search space, too much constraint in scientific exploration may also be bad for progress; it may therefore be helpful for scientists to operationalize theory as both an a priori filter and a tool for post hoc ‘compression’ and synthesis.

For students and early-career researchers, balancing exploration and abstraction can be difficult to navigate. Outside explicit theoretical biology laboratories or programs, training in biology often emphasizes knowledge of experimental design, memorization of known biological processes, and proficiency in basic data analysis. While quantitative and computational rigor are increasingly relevant in the era of computational biology and genomics, and in what we propose as a new theory-centric biology, these skills are not typically part of mainstream biology curricula. Presently, mathematical and/or conceptual synthesis are often left largely unacknowledged, rendering theory generation an ineffable, almost fantastical practice, rather than a way of thinking or doing science that can be learned. Yet the ability to move between iterative empirical investigation and theory-driven synthesis may be precisely what the current moment demands.

The challenge extends beyond individual scientists to the structure of scientific communication itself. As the volume of biological research continues to grow, no single researcher can comprehensively track the expanding space of competing hypotheses and explanations, leaving potentially important theoretical connections fragmented across subdisciplines. If theory is to assume a larger role in biology, hypotheses and theoretical claims may need to become more accessible to systematic analysis through machine-readable representations (Booeshaghi et al., 2026) that allow both scientists and computational agents to reason across competing ideas. Likewise, preserving space for theoretical reasoning within scientific publications remains essential. Introduction and discussion sections provide a venue for articulating hypotheses, interpreting observations, and situating results within broader conceptual frameworks, but in many cases, have become pithy data summaries designed to meet journal formatting requirements. Future computational tools may help navigate this space, but only if the scientific record continues to explicitly capture the diversity of explanations that drive biological discovery.

Biology has never advanced by data alone. Darwin’s theory of natural selection and Schrödinger’s vision of an information-bearing molecule did not emerge spontaneously from experimental data, but from attempts to make sense of existing observations by reconciling extant principles while also developing new ones. Today’s genomic and cellular atlases are arguably richer than anything available to previous generations. Whether they provide the substrate for comparable conceptual revolutions or are simply food for black-box predictors will depend not only on how much we measure, but on how boldly we theorize.

## References

[bib1] Adduri AK, Gautam D, Bevilacqua B, Imran A, Shah R, Naghipourfar M, Teyssier N, Ilango R, Nagaraj S, Dong M, Ricci-Tam C, Carpenter C, Subramanyam V, Winters A, Tirukkovular S, Sullivan J, Plosky BS, Eraslan B, Youngblut ND, Leskovec J, Gilbert LA, Konermann S, Hsu PD, Dobin A, Burke DP, Goodarzi H, Roohani YH (2025). Predicting cellular responses to perturbation across diverse contexts with state. bioRxiv.

[bib2] Avsec Ž, Agarwal V, Visentin D, Ledsam JR, Grabska-Barwinska A, Taylor KR, Assael Y, Jumper J, Kohli P, Kelley DR (2021). Effective gene expression prediction from sequence by integrating long-range interactions. Nature Methods.

[bib3] Booeshaghi AS, Luebbert L, Pachter L (2026). Science should be machine-readable. bioRxiv.

[bib4] Breiman L (2001). Random forests. Machine Learning.

[bib5] Brixi G, Durrant MG, Ku J, Naghipourfar M, Poli M, Sun G, Brockman G, Chang D, Fanton A, Gonzalez GA, King SH, Li DB, Merchant AT, Nguyen E, Ricci-Tam C, Romero DW, Schmok JC, Taghibakhshi A, Vorontsov A, Yang B, Deng M, Gorton L, Nguyen N, Wang NK, Pearce MT, Simon E, Adams E, Amador ZJ, Ashley EA, Baccus SA, Dai H, Dillmann S, Ermon S, Guo D, Herschl MH, Ilango R, Janik K, Lu AX, Mehta R, Mofrad MRK, Ng MY, Pannu J, Ré C, St John J, Sullivan J, Tey J, Viggiano B, Zhu K, Zynda G, Balsam D, Collison P, Costa AB, Hernandez-Boussard T, Ho E, Liu M-Y, McGrath T, Powell K, Pinglay S, Burke DP, Goodarzi H, Hsu PD, Hie BL (2026). Genome modelling and design across all domains of life with Evo 2. Nature.

[bib6] Cheong R, Rhee A, Wang CJ, Nemenman I, Levchenko A (2011). Information transduction capacity of noisy biochemical signaling networks. Science.

[bib7] Chuai G, Chen X, Yang X, Zhang C, Qu K, Wang Y, Li W, Yang J, Si D, Xing F, Gao Y, Wu S, Fu S, He B, Liu Q (2026). Towards building a world model to simulate perturbation-induced cellular dynamics by AlphaCell. bioRxiv.

[bib8] Darwin C (1859). On the Origin of Species.

[bib9] Dibaeinia P, Babu S, Knudson M, ElSheikh A, Wen Y, Liu H, Perera J, Khan AA (2026). Virtual cells need context, not just scale. bioRxiv.

[bib10] Dubova M, Moskvichev A, Zollman K (2022). Against theory-motivated experimentation in science. MetaArXiv.

[bib11] Godfrey-Smith P (2021). Theory and Reality: An Introduction to the Philosophy of Science.

[bib12] Hempel C (1966). Philosophy of Natural Science.

[bib13] Jacob F (1988). The Statue Within: An Autobiography.

[bib14] Judson HF (2011). The Eighth Day of Creation: Makers of the Revolution in Biology.

[bib15] Kay LE (2000). Who Wrote the Book of Life? A History of the Genetic Code.

[bib16] Kitcher P, Kitcher P (1989). Scientific Explanation.

[bib17] Kitcher P (2003). In Mendel’s Mirror: Philosophical Reflections on Biology.

[bib18] Kuhn T (1962). The Structure of Scientific Revolutions.

[bib19] Liang T, Singh E, Parhi R, Cloninger A, Wang YX (2026). The inductive bias of convolutional neural networks: locality and weight sharing reshape implicit regularization. arXiv.

[bib20] Linder J, Srivastava D, Yuan H, Agarwal V, Kelley DR (2025). Predicting RNA-seq coverage from DNA sequence as a unifying model of gene regulation. Nature Genetics.

[bib21] Mayr E (1982). The Growth of Biological Thought: Diversity, Evolution, and Inheritance.

[bib22] Nagel E (1961). The Structure of Science: Problems in the Logic of Scientific Explanation.

[bib23] Nguyen E, Poli M, Durrant MG, Kang B, Katrekar D, Li DB, Bartie LJ, Thomas AW, King SH, Brixi G, Sullivan J, Ng MY, Lewis A, Lou A, Ermon S, Baccus SA, Hernandez-Boussard T, Ré C, Hsu PD, Hie BL (2024). Sequence modeling and design from molecular to genome scale with Evo. Science.

[bib24] Nurse P (2021). Biology must generate ideas as well as data. Nature.

[bib25] Pearl J (1986). Fusion, propagation, and structuring in belief networks. Artificial Intelligence.

[bib26] Popper K (1992). The Logic of Scientific Discovery.

[bib27] Quake SR (2024). The cellular dogma. Cell.

[bib28] Schneider TD, Stormo GD, Gold L, Ehrenfeucht A (1986). Information content of binding sites on nucleotide sequences. Journal of Molecular Biology.

[bib29] Schneider TD (2006). Claude Shannon: biologist. The founder of information theory used biology to formulate the channel capacity. IEEE Engineering in Medicine and Biology Magazine.

[bib30] Schrödinger E (1944). What Is Life? The Physical Aspect of the Living Cell.

[bib31] Shannon CE (1948). A mathematical theory of communication. Bell System Technical Journal.

[bib32] Shmueli G (2010). To Explain or to Predict?. Statistical Science.

[bib33] Souza H, Mehta P (2026). Parameter-free representations outperform single-cell foundation models on downstream benchmarks. bioRxiv.

[bib34] Svensson V, Khan U, Heydari H, Ubas AA, Thomas N, Merico D, Goodarzi H, Yu J, Alidoust N, Gandhi S (2026). Back to basics: observed statistics are sufficient to predict drug responses. bioRxiv.

[bib35] Tang Y, Hoffmann A (2022). Quantifying information of intracellular signaling: progress with machine learning. Reports on Progress in Physics. Physical Society.

[bib36] Theodoris CV, Xiao L, Chopra A, Chaffin MD, Al Sayed ZR, Hill MC, Mantineo H, Brydon EM, Zeng Z, Liu XS, Ellinor PT (2023). Transfer learning enables predictions in network biology. Nature.

[bib37] Uda S (2020). Application of information theory in systems biology. Biophysical Reviews.

[bib38] Vaswani A, Brain G, Shazeer N, Parmar N, Uszkoreit J, Jones L, Gomez AN, Kaiser Ł, Polosukhin I (2023). Attention is all you need. arXiv.

[bib39] Watson JL, Juergens D, Bennett NR, Trippe BL, Yim J, Eisenach HE, Ahern W, Borst AJ, Ragotte RJ, Milles LF, Wicky BIM, Hanikel N, Pellock SJ, Courbet A, Sheffler W, Wang J, Venkatesh P, Sappington I, Torres SV, Lauko A, De Bortoli V, Mathieu E, Ovchinnikov S, Barzilay R, Jaakkola TS, DiMaio F, Baek M, Baker D (2023). De novo design of protein structure and function with RFdiffusion. Nature.

[bib40] Woodward J (2003). Making Things Happen: A Theory of Causal Explanation.

[bib41] Yanai I, Lercher M (2019). Night science. Genome Biology.

[bib42] Yanai I, Lercher M (2020). A hypothesis is a liability. Genome Biology.

